# Methyl­ene bis­(dithio­benzoate)

**DOI:** 10.1107/S1600536808037379

**Published:** 2008-11-13

**Authors:** Yao-Ren Liang, Hung-Chun Tong, Yih-Hsing Lo, Chia-Her Lin, Ting Shen Kuo

**Affiliations:** a Department of Chemical Engineering, Tatung University, Taipei 104, Taiwan; b Department of Chemistry, Chung-Yuan Christian University, Chung-Li 320, Taiwan; c Department of Chemistry, National Normal Taiwan University, Taipei 106, Taiwan

## Abstract

In the title compound, C_15_H_12_S_4_, two phenyl­dithio­carboxyl­ate units are linked through a methyl­ene C atom on a twofold rotation axis. The central S—CH_2_—S angle of 116.9 (5)° is significantly larger than the ideal tetra­hedral value. The dihedral angle formed by the two phenyl rings is 68.2 (1)°. The refined Flack parameter of 0.2 (3) does not permit unambiguous determination of the absolute structure.

## Related literature

For related structures, see: Shrivastav *et al.* (2002 ▶); Gonzalez-Castro *et al.* (2000 ▶); Quintanilla *et al.* (2005 ▶).
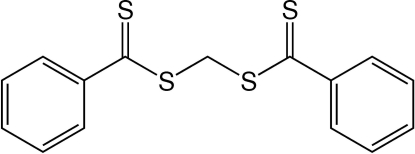


## Experimental

### 

#### Crystal data

C_15_H_12_S_4_
*M* *_r_* = 320.49
Orthorhombic, 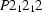

*a* = 11.5800 (3) Å
*b* = 14.6440 (11) Å
*c* = 4.2710 (7) Å
*V* = 724.27 (13) Å^3^
*Z* = 2
Mo *K*α radiation
μ = 0.64 mm^−1^
*T* = 200 (2) K
0.11 × 0.08 × 0.02 mm

#### Data collection

Nonius KappaCCD diffractometer
Absorption correction: multi-scan (*SORTAV*; Blessing, 1995 ▶) *T*_min_ = 0.933, *T*_max_ = 0.987
5256 measured reflections
1317 independent reflections
931 reflections with *I* > 2σ(*I*)
*R*_int_ = 0.096

#### Refinement

*R*[*F* ^2^ > 2σ(*F* ^2^)] = 0.056
*wR*(*F* ^2^) = 0.157
*S* = 1.13
1317 reflections
87 parameters
H-atom parameters constrained
Δρ_max_ = 0.37 e Å^−3^
Δρ_min_ = −0.46 e Å^−3^
Absolute structure: Flack (1983 ▶), 505 Friedel pairs
Flack parameter: 0.2 (3)

### 

Data collection: *COLLECT* (Nonius, 1999 ▶); cell refinement: *DENZO* and *SCALEPACK* (Otwinowski & Minor, 1997 ▶); data reduction: *DENZO* (Otwinowski & Minor, 1997 ▶) and *SCALEPACK*; program(s) used to solve structure: *SHELXS97* (Sheldrick, 2008 ▶); program(s) used to refine structure: *SHELXL97* (Sheldrick, 2008 ▶); molecular graphics: *SHELXTL* (Sheldrick, 2008 ▶); software used to prepare material for publication: *SHELXTL*.

## Supplementary Material

Crystal structure: contains datablocks I, global. DOI: 10.1107/S1600536808037379/bi2313sup1.cif

Structure factors: contains datablocks I. DOI: 10.1107/S1600536808037379/bi2313Isup2.hkl

Additional supplementary materials:  crystallographic information; 3D view; checkCIF report

## Figures and Tables

**Fig. 1.**
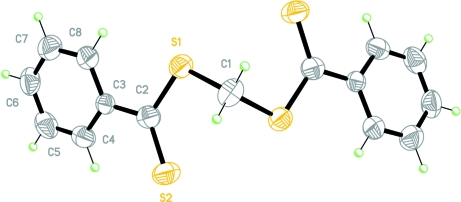
Molecular structure of the title compound showing displacement ellipsoids at the 50% probability level for non-H atoms. Non-labelled atoms are related to labelled atoms by the symmetry code 1-x, 2-y, z.

## supplementary crystallographic information

### Comment

During studies on the reactivity of the RuS_2_ complex {Ru(Tp)(PPh_3_)[S_2_CC_6_H_5_]}, (hydridotripyrazol-1-ylborato-κ^3^*N*^2^,*N*^2^',*N*^2^'')(phenyldithiocarboxylato-κ^2^*S*,*S*')(triphenylphosphine-*κP*)ruthenium, with CH_3_CN in dichloromethane, we unexpectedly obtained crystals of the title compound. It consists of two phenyldithiocarboxylate units bridged by a methylene group. The ^1^H NMR spectrum in CDCl_3_ shows one singlet at 5.31 ppm, assignable to SCH_2_S. The EI mass spectrum shows the molecular ion [C_15_H_12_S_4_]^+^ with the characteristic isotopic distribution patterns. In the crystal, the C2—S2 bond length of 1.643 (6) Å is slightly longer than expected for a C=S double bond (*ca* 1.61 Å), while the C2—S1 and C1—S1 distances of 1.743 (6) and 1.794 (5) Å, respectively, are clearly single bonds. The S—C—S angle of 116.9 (5)° is larger than the ideal tetrahedral value, probably due to repulsion between the CS_2_ groups.

### Experimental

The title compound was obtained unexpectedly during studies on the reactivity of {Ru(Tp)(PPh_3_)[S_2_CC_6_H_5_]} with CH_3_CN in dichloromethane. To a solution of {Ru(Tp)(PPh_3_)[S_2_CC_6_H_5_]} (2.00 g, 2.73 mmol) in CH_2_Cl_2_ (20 ml), an excess of CH_3_CN (2 ml) was added. The resulting yellow solution was heated to reflux for 3 h and the yellow precipitate obtained was filtered and washed with methanol and water to remove excess reagents. The compound was then dried under vacuum to give 0.83 g (91% yield). Crystals for X-ray structure analysis were obtained by recrystallization of the crude product from dichloromethane–hexane.

Elemental analysis calculated: C, 56.21; H, 3.77%; found: C, 56.19; H, 3.69%. ^1^H NMR (CDCl_3_,303 K, ppm): δ 7.98–7.35 (m, 10H, Ph), 5.31 (s, 2H, CH_2_). MS (*m*/*z*): 320.5 (*M*^+^).

### Refinement

H atoms were placed in idealized positions and constrained to ride on their parent atoms, with C—H = 0.95–0.96 Å and *U*_iso_(H) = 1.2*U*_eq_(C). The refined Flack parameter of 0.2 (3) does not permit unambiguous determination of the absolute structure.

### Crystal data

**Table:**

C_15_H_12_S_4_	*F*_000_ = 332
*M**_r_* = 320.49	*D*_x_ = 1.470 Mg m^−^^3^
Orthorhombic, *P*2_1_2_1_2	Mo *K*α radiation λ = 0.71073 Å
Hall symbol: P 2 2ab	Cell parameters from 5256 reflections
*a* = 11.5800 (3) Å	θ = 2.0–25.4º
*b* = 14.6440 (11) Å	µ = 0.64 mm^−^^1^
*c* = 4.2710 (7) Å	*T* = 200 (2) K
*V* = 724.27 (13) Å^3^	Plate, yellow
*Z* = 2	0.11 × 0.08 × 0.02 mm

### Data collection

**Table:**

Nonius KappaCCD diffractometer	1317 independent reflections
Radiation source: fine-focus sealed tube	931 reflections with *I* > 2σ(*I*)
Monochromator: graphite	*R*_int_ = 0.096
*T* = 200(2) K	θ_max_ = 25.5º
φ scans	θ_min_ = 2.2º
Absorption correction: multi-scan(SORTAV; Blessing, 1995)	*h* = −13→13
*T*_min_ = 0.933, *T*_max_ = 0.987	*k* = −17→17
5256 measured reflections	*l* = −5→4

### Refinement

**Table:**

Refinement on *F*^2^	Hydrogen site location: inferred from neighbouring sites
Least-squares matrix: full	H-atom parameters constrained
*R*[*F*^2^ > 2σ(*F*^2^)] = 0.056	*w* = 1/[σ^2^(*F*_o_^2^) + (0.0733*P*)^2^] where *P* = (*F*_o_^2^ + 2*F*_c_^2^)/3
*wR*(*F*^2^) = 0.157	(Δ/σ)_max_ < 0.001
*S* = 1.13	Δρ_max_ = 0.37 e Å^−^^3^
1317 reflections	Δρ_min_ = −0.46 e Å^−^^3^
87 parameters	Extinction correction: none
Primary atom site location: structure-invariant direct methods	Absolute structure: Flack (1983), 505 Friedel pairs
Secondary atom site location: difference Fourier map	Flack parameter: 0.2 (3)

### Special details

**Table:**

Geometry. All esds (except the esd in the dihedral angle between two l.s. planes) are estimated using the full covariance matrix. The cell esds are taken into account individually in the estimation of esds in distances, angles and torsion angles; correlations between esds in cell parameters are only used when they are defined by crystal symmetry. An approximate (isotropic) treatment of cell esds is used for estimating esds involving l.s. planes.
Refinement. Refinement of *F*^2^ against ALL reflections. The weighted *R*-factor *wR* and goodness of fit *S* are based on *F*^2^, conventional *R*-factors *R* are based on *F*, with *F* set to zero for negative *F*^2^. The threshold expression of *F*^2^ > σ(*F*^2^) is used only for calculating *R*-factors(gt) *etc*. and is not relevant to the choice of reflections for refinement. *R*-factors based on *F*^2^ are statistically about twice as large as those based on *F*, and *R*- factors based on ALL data will be even larger.

### Fractional atomic coordinates and isotropic or equivalent isotropic displacement parameters (Å^2^)

**Table:**

	*x*	*y*	*z*	*U*_iso_*/*U*_eq_	
S1	0.47264 (14)	0.89784 (9)	0.5726 (3)	0.0446 (5)	
S2	0.72612 (15)	0.92562 (10)	0.5091 (5)	0.0613 (6)	
C1	0.5000	1.0000	0.792 (2)	0.049 (2)	
H1A	0.4346	1.0109	0.9253	0.058*	
C2	0.6098 (5)	0.8635 (4)	0.4525 (14)	0.0437 (15)	
C3	0.6104 (5)	0.7734 (4)	0.2900 (15)	0.0381 (14)	
C4	0.7071 (5)	0.7447 (4)	0.1238 (15)	0.0480 (17)	
H4A	0.7733	0.7828	0.1147	0.058*	
C5	0.7082 (6)	0.6617 (4)	−0.0279 (16)	0.0572 (18)	
H5A	0.7749	0.6428	−0.1397	0.069*	
C6	0.6127 (6)	0.6064 (4)	−0.0173 (15)	0.0562 (18)	
H6A	0.6136	0.5491	−0.1211	0.067*	
C7	0.5161 (6)	0.6336 (4)	0.1422 (16)	0.0600 (19)	
H7A	0.4503	0.5949	0.1492	0.072*	
C8	0.5140 (6)	0.7159 (4)	0.2913 (15)	0.0499 (17)	
H8A	0.4459	0.7345	0.3977	0.060*	

### Atomic displacement parameters (Å^2^)

**Table:**

	*U*^11^	*U*^22^	*U*^33^	*U*^12^	*U*^13^	*U*^23^
S1	0.0456 (9)	0.0410 (8)	0.0472 (9)	−0.0042 (7)	0.0029 (7)	0.0005 (7)
S2	0.0472 (10)	0.0568 (9)	0.0800 (14)	−0.0132 (7)	−0.0145 (9)	0.0109 (10)
C1	0.063 (7)	0.043 (5)	0.039 (5)	−0.008 (4)	0.000	0.000
C2	0.042 (4)	0.048 (3)	0.041 (3)	−0.003 (2)	−0.012 (3)	0.017 (3)
C3	0.035 (3)	0.035 (3)	0.044 (4)	0.001 (3)	0.000 (3)	0.016 (3)
C4	0.038 (4)	0.058 (4)	0.048 (4)	0.002 (3)	0.002 (3)	0.018 (3)
C5	0.055 (4)	0.062 (4)	0.054 (4)	0.019 (3)	0.016 (4)	0.011 (4)
C6	0.068 (5)	0.039 (3)	0.062 (4)	0.013 (3)	0.012 (4)	−0.001 (4)
C7	0.057 (5)	0.046 (3)	0.077 (5)	−0.001 (3)	0.017 (4)	−0.005 (3)
C8	0.044 (4)	0.042 (3)	0.064 (4)	0.003 (3)	0.008 (4)	0.001 (3)

### Geometric parameters (Å, °)

**Table:**

S1—C2	1.743 (6)	C4—H4A	0.950
S1—C1	1.794 (5)	C5—C6	1.370 (9)
S2—C2	1.643 (6)	C5—H5A	0.950
C1—S1^i^	1.794 (5)	C6—C7	1.369 (8)
C1—H1A	0.960	C6—H6A	0.950
C2—C3	1.491 (8)	C7—C8	1.364 (8)
C3—C4	1.391 (8)	C7—H7A	0.950
C3—C8	1.399 (8)	C8—H8A	0.950
C4—C5	1.377 (9)		
			
C2—S1—C1	103.5 (2)	C6—C5—C4	119.9 (6)
S1—C1—S1^i^	116.9 (5)	C6—C5—H5A	120.1
S1—C1—H1A	108.0	C4—C5—H5A	120.1
S1^i^—C1—H1A	108.0	C7—C6—C5	120.3 (6)
C3—C2—S2	123.6 (4)	C7—C6—H6A	119.9
C3—C2—S1	113.4 (4)	C5—C6—H6A	119.9
S2—C2—S1	123.0 (4)	C8—C7—C6	120.2 (7)
C4—C3—C8	117.5 (6)	C8—C7—H7A	119.9
C4—C3—C2	120.6 (5)	C6—C7—H7A	119.9
C8—C3—C2	121.8 (5)	C7—C8—C3	121.1 (6)
C5—C4—C3	121.0 (6)	C7—C8—H8A	119.5
C5—C4—H4A	119.5	C3—C8—H8A	119.5
C3—C4—H4A	119.5		
			
C2—S1—C1—S1^i^	78.0 (2)	C2—C3—C4—C5	−179.7 (5)
C1—S1—C2—C3	174.1 (4)	C3—C4—C5—C6	0.3 (9)
C1—S1—C2—S2	−6.7 (5)	C4—C5—C6—C7	0.3 (10)
S2—C2—C3—C4	−12.4 (8)	C5—C6—C7—C8	0.2 (10)
S1—C2—C3—C4	166.7 (5)	C6—C7—C8—C3	−1.3 (10)
S2—C2—C3—C8	169.3 (5)	C4—C3—C8—C7	1.8 (9)
S1—C2—C3—C8	−11.6 (7)	C2—C3—C8—C7	−179.8 (6)
C8—C3—C4—C5	−1.4 (9)		

Symmetry codes: (i) −*x*+1, −*y*+2, *z*.
